# Epitope mapping and characterization of a novel Nsp10-specific monoclonal antibody that differentiates genotype 2 PRRSV from genotype 1 PRRSV

**DOI:** 10.1186/s12985-017-0782-9

**Published:** 2017-06-19

**Authors:** Zhibang Zhang, Xuexia Wen, Jianguo Dong, Xinna Ge, Lei Zhou, Hanchun Yang, Xin Guo

**Affiliations:** Key Laboratory of Animal Epidemiology and Zoonosis of the Ministry of Agriculture, College of Veterinary Medicine and State Key Laboratory of Agrobiotechnology, China Agricultural University, No. 2 Yuanmingyuan West Road, Haidian District, Beijing, 100193 People’s Republic of China

**Keywords:** Porcine reproductive and respiratory syndrome virus (PRRSV), Non-structural protein 10 (Nsp10), Monoclonal antibody (mAb), B-cell epitope, Differential diagnosis

## Abstract

**Background:**

Porcine reproductive and respiratory syndrome virus (PRRSV), the causative agent of PRRS, has two distinct and highly diverse genotypes (genotype 1 and genotype 2) in the field. Accurate diagnosis and differentiation of the two genotypes of PRRSV are critical to the effective prevention and control of PRRS. The non-structural protein 10 (Nsp10) plays a vital role in viral replication and is one of the most conserved proteins of PRRSV, thus constituting a good candidate for PRRSV diagnosis.

**Results:**

In this study, we generated a monoclonal antibody (mAb) 4D9 against Nsp10 by immunizing BALB/c mice with purified recombinant Nsp10 expressed by an *Escherichia coli* system. Through fine epitope mapping of mAb 4D9 using a panel of eukaryotic expressed polypeptides with GFP-tags, we identified the motif ^286^AIQPDYRDKL^295^ as the minimal unit of the linear B-cell epitope recognized by mAb 4D9. Protein sequence alignment indicated that ^286^AIQPDYRDKL^295^ was highly conserved in genotype 2 PRRSV strains, whereas genotype 1 PRRSV strains had variable amino acids in this motif. Furthermore, a mutant of the motif carrying two constant amino acids of genotype 1 PRRSV, Cys290 and Glu293, failed to react with mAb 4D9. More importantly, the mAb 4D9 could differentiate genotype 2 PRRSV strains from genotype 1 PRRSV strains using Western blotting and immunofluorescence analysis.

**Conclusion:**

Our findings suggest that Nsp10-specific mAb generated in this study could be a useful tool for basic research and may facilitate the establishment of diagnostic methods to discriminate between genotype 1 and genotype 2 PRRSV infection.

## Background

Porcine reproductive and respiratory syndrome (PRRS) is one of the most important viral diseases of pigs worldwide, causing annual economic losses of about US $664 million to the US swine industry [1]. PRRS is characterized by reproductive failure in pregnant sows and respiratory disorders in all age pigs [2]. The causative agent, porcine reproductive and respiratory syndrome virus (PRRSV), is an enveloped, single-stranded RNA (+) virus belonging to the order *Nidovirales*, family *Arteriviridae* [3]. PRRSV is categorized into two genotypes based on the genetic diversity. Genotype 1 (European) and genotype 2 (North American) share only ~65% nucleotide identity at the genomic level [4, 5]. In the field, the virus evolves rapidly and shows an extensive genetic heterogeneity and antigenic variability, which makes accurate diagnosis and control of PRRS very difficult [6].

The PRRSV RNA genome is about 15 kb in length, containing at least 11 open reading frames (ORFs) [7]. The ORF1a and ORF1b encode replication-related non-structural proteins (Nsps), whereas ORFs 2–7 are translated from a nested set of subgenomic RNA (sgRNA) encoding the structural proteins [8, 9]. PRRSV Nsp10 lies in ORF1b region and encodes helicase [10], which possesses ATPase activity and can unwind dsRNA [11]. A recent study revealed that PRRSV Nsp10 could bind both ssDNA and dsDNA, and mutations at Cys25 and His32 abolished the binding and unwinding activity of Nsp10 [12]. The recent studies have demonstrated that PRRSV Nsp10 could induce apoptosis through both extrinsic and mitochondria-dependent pathways [13], and the Nsp9- and Nsp10-coding regions of highly pathogenic PRRSV contributed to its fatal virulence in piglets [14]. However, there is little knowledge about the epitope mapping of PRRSV Nsp10. In this study, we generated a PRRSV Nsp10-specific mAb, fine mapped its epitope and demonstrated that it can differentiate the Nsp10 of the genotype 2 from that of genotype 1.

## Results

### Expression and purification of recombinant Nsp10 in *E. coli*

**Fig. 1:**
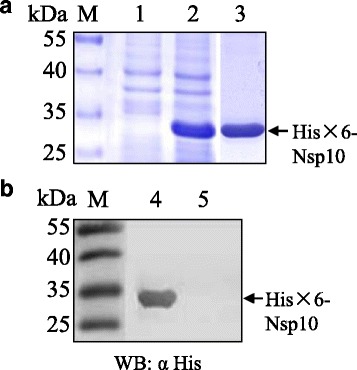
Analysis of expressed recombinant Nsp10 by SDS-PAGE (**a**) and Western blotting (**b**) with anti-His mAb. Lane M: protein molecular weight marker; Lane 1: lysates of pET-28a-Nsp10 transformed *E. coli* BL21 (DE3) before IPTG induction; Lane 2: lysates of pET-28a-Nsp10 transformed *E. coli* BL21 (DE3) after IPTG induction; Lane 3 and 4: purified recombinant Nsp10; Lane 5: lysates of pET-28a transformed *E. coli* BL21 (DE3) as negative control

The recombinant protein His × 6-Nsp10 was expressed in *E. coli* with an expected molecular weight of approximately 32 kDa (Fig. 1a). Since the recombinant protein was presented predominantly in an insoluble form (inclusion bodies), we purified the protein by excising the gel piece that contained the protein His × 6-Nsp10 from the SDS-PAGE gel. Then we determined the purity of the prepared recombinant His × 6-Nsp10 with SDS-PAGE (Fig. 1a). Western blotting analysis showed that the purified His × 6-Nsp10 protein could be recognized by anti-His Tag mAb (Fig. 1b). The results indicated that the purified recombinant His × 6-Nsp10 had good reactivity and was suitable for immunization.

### Production and characterization of Nsp10-specific mAb

**Fig. 2:**
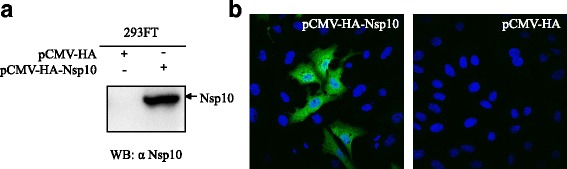
Specific reactivity of Nsp10-specific mAb with the eukaryotic expressed Nsp10. **a** Western blotting of 293FT cells transfected with eukaryotic recombinant plasmids pCMV-HA-Nsp10 or the empty vector pCMV-HA. **b** Immunofluorescence staining of Vero cells transfected with eukaryotic plasmids pCMV-HA-Nsp10 (left) or pCMV-HA (right)

Hybridomas were screened by testing the supernatants with PRRSV Nsp10-specific indirect ELISA. One hybridoma cell line secreting the antibodies specific against Nsp10 was selected and subcloned thrice by limiting dilution. Isotype determination showed that Nsp10-specific mAb 4D9 is a subclass IgG1/κ-type. To further determine the specificity of the mAb, the pCMV-Nsp10 plasmid transfected cells were analyzed by Western blotting and confocal microscopy using the mAb 4D9 as the primary antibody. The results of Western blotting revealed that the mAb 4D9 could specifically react with eukaryotic expressed Nsp10 protein but not with the empty plasmid pCMV-HA transfected samples (Fig. 2a). Confocal microscopy showed a brilliant fluorescence staining in pCMV-HA-Nsp10 transfected cells only, and Nsp10 protein located in the cytoplasm of Vero cells (Fig. 2b). Those results demonstrated that the generated mAb 4D9 is specific for PRRSV Nsp10. The in vitro neutralization test showed that the mAb 4D9 is not a neutralizing antibody (data not shown).

### Precise localization of mAb 4D9 epitope

**Fig. 3:**
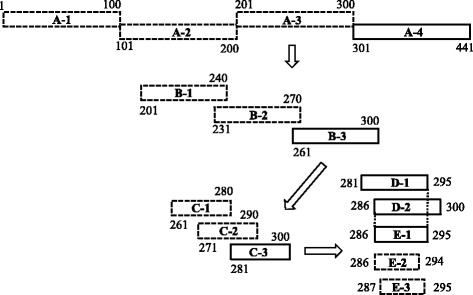
Schematic diagram showing the fragments of Nsp10 protein and their relative positions. Bars represent truncated Nsp10 peptides. The names of the peptides are shown in *bold letters*. Numbers represent amino acid positions of the peptides in Nsp10. Peptides that were negative in Western blotting against mAb 4D9 are shown by bars with *dash lines*, and peptides that were positive are shown by bars with *solid lines*

**Fig. 4:**
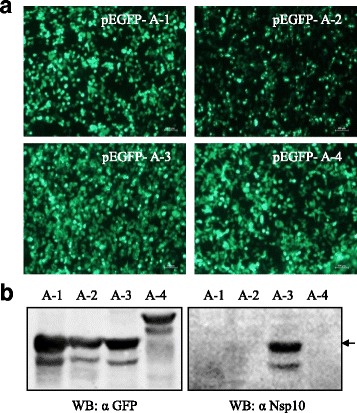
Primary scanning of the B-cell epitope of Nsp10-specific mAb. **a** The expression of the fragments of Nsp10 (A-1, A-2, A-3 and A-4) was visualized by fluorescent microscopy in recombinant plasmids transfected 293FT cells. **b** The reactivity of the four fragments with Nsp10-specific mAb and anti-GFP polyclonal antibodies were tested by Western blotting

**Fig. 5:**
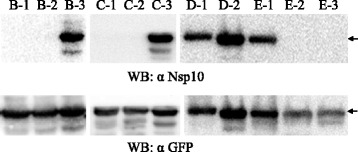
Fine mapping of the mAb 4D9 epitope in Nsp10. Recombinant plasmids expressing a series of truncated Nsp10 were transfected into 293FT cells, and the expressed peptides were probed with anti-GFP polyclonal antibodies and the mAb 4D9 by Western blotting

Four peptide fragments covering the full-length Nsp10 without overlapping regions (Fig. 3) were expressed as GFP-fusion proteins by transfecting 293FT cells respectively. The expression of all GFP-fusion proteins was confirmed by visualizing the green fluorescence in transfected 293FT cells (Fig. 4a). Western blotting results showed that all fragments reacted with the anti-GFP polyclonal antibodies. However, only the fragment A-3 could be recognized by the Nsp10-specific mAb 4D9 (Fig. 4b), indicating that this region contains the epitope that binds with mAb 4D9. Then, the fragment A-3 was divided into three overlapping parts and each with a length of 40 amino acids (Fig. 3). Western blotting analysis indicated that the third part, B-3, contains the mAb 4D9 epitope (Fig. 5). Next, B-3 was divided into three overlapping segments and C-3 was proved to be a segment comprising mAb 4D9 epitope (Fig. 5). The segment C-3 is 20 amino acids in length, and it was divided into two sections further. The two sections were 15 amino acids in length with 10 amino acid overlapping with each other (Fig. 3). Both sections could react with the mAb 4D9 in Western blotting (Fig. 5). It suggested that the overlapping region, AIQPDYRDKL, contains the mAb 4D9 epitope. To determine the core sequences of the epitope, we tested the reaction of mAb 4D9 with peptides that had the amino acid deleted from the motif AIQPDYRDKL one by one from its N- and C-termini respectively (Fig. 3). The results showed that even single amino acid deletion from either end of the motif AIQPDYRDKL deprived its reaction with the mAb 4D9 (Fig. 5), suggesting that the motif AIQPDYRDKL was the minimal unit of the epitope required for binding with the mAb 4D9.

### Homology analysis of the identified Nsp10 epitope in different PRRSV strains

**Fig. 6:**
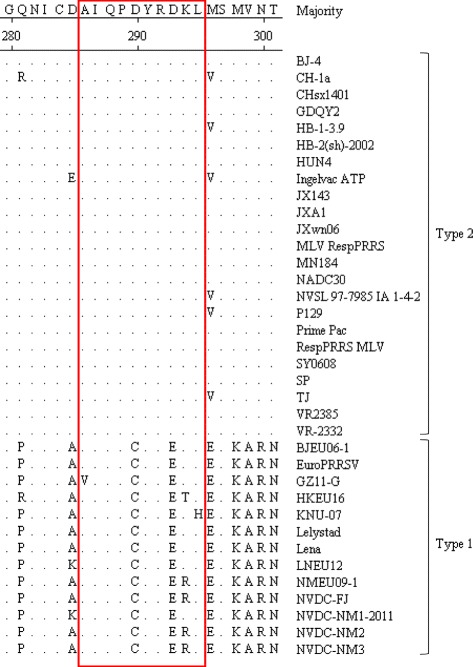
Alignment of PRRSV Nsp10 protein of genotype 1 and genotype 2 isolates. The epitope-containing region is boxed. Dots represent residues that are identical to the majority

To evaluate if the linear epitope recognized by the mAb 4D9 is conserved among PRRSV isolates, we performed sequence alignment with genotype 1 and genotype 2 PRRSV Nsp10. The results indicated that the epitope ^286^AIQPDYRDKL^295^ is highly conserved among genotype 2 PRRSV isolates (Fig. 6), suggesting that mAb 4D9 can recognize all genotype 2 strains of PRRSV. However, there are some substitutions in the corresponding region in genotype 1 PRRSV isolates compared with the epitope in genotype 2 PRRSV isolates (Fig. 6). Of note, there are two constant amino acid variations in genotype 1 PRRSV compared with genotype 2 PRRSV. One is a D to C variation at site 290 and the other is a D to E variation at position 293.

### Mutation analysis of the epitope

**Fig. 7:**
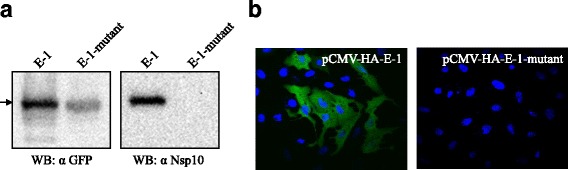
Reactivity of mAb 4D9 with the mutant of the epitope ^286^AIQPDYRDKL^295^. The plasmid pCMV-HA-E-1 expressing the epitope (E-1) and pCMV-HA-E-1-mutant expressing a mutant of E-1 with 2 mutations (^290^D/C^290^, ^293^D/E^293^) were constructed and transfected into Vero cells. The reaction of mAb 4D9 with the two epitopes was tested. **a** Western blotting of lysates of Vero cells transfected with pCMV-HA-E-1or pCMV-HA- E-1-mutant. **b** Confocal microscopy of Vero cells transfected with pCMV-HA-E-1 or pCMV-HA-E-1-mutant

To determine if the variation between genotype1 and genotype 2 PRRSV Nsp10 changed their binding ability to the mAb 4D9, we constructed a mutant epitope ^286^AIQPCYREKL^295^ carrying the two mutations in mAb 4D9 epitope and tested if it can react with the mAb 4D9. Both Western blotting analysis and confocal microscopy showed that the mAb 4D9 failed to react with the mutant epitope ^286^AIQPCYREKL^295^, indicating that alteration of the two amino acids affected the reactivity of the epitope with the mAb 4D9 (Fig. 7).

### The mAb 4D9 could differentiate genotype 2 PRRSV from genotype 1 PRRSV

**Fig. 8:**
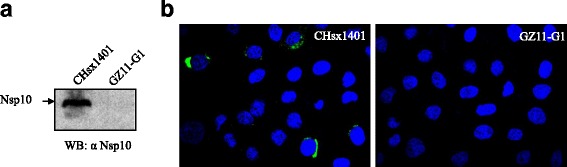
Detection of Nsp10 with mAb 4D9 in genotype 1 or genotype 2 PRRSV-infected MARC-145 cells. **a** Western blotting of the lysates of PRRSV-infected MARC-145 cells using mAb 4D9 as primary antibodies. **b** Confocal microscopy of PRRSV-infected MARC-145 cells staining with mAb 4D9

Since the two amino acids, Cys290 and Glu293 in the mAb 4D9 epitope are conserved among all known genotype 1 PRRSV isolates, we speculated that the mAb 4D9 could not recognize the Nsp10 of genotype 1 PRRSV isolates. To verify our hypothesis, a genotype 1 PRRSV strain, GZ11-G1, encompassing the motif ^286^VIQPCYREKL^295^ and a genotype 2 strain, CHsx1401, containing the motif ^286^AIQPDYRDKL^295^ were probed with the Nsp10-specific mAb by Western blotting and confocal microscopy. Western blotting analysis showed that mAb 4D9 could recognize the Nsp10 protein of CHsx1401 but not that of GZ11-G1 (Fig. 8a). The results of confocal microscopy revealed that fluorescence signal against Nsp10 was only observed in CHsx1401-infected cells (Fig. 8b). It indicated that the mAb 4D9 generated in this study could serve as a useful tool to differentiate genotype 2 PRRSV from genotype 1 PRRSV.

## Discussion

The emergence of genotype 1 PRRSV in China recently complicated the control of PRRS caused by genotype 2 PRRSV [15]. The coexistence of two genotypes of PRRSV in swine industry makes the accurate diagnosis more critical for the control and prevention of PRRS.

The molecular diagnosis of infection with PRRSV is hampered by its tremendous strain diversity. Even within each genotype, the genetic variation among PRRSV strains is substantial [6]. In the present study, we focused on the Nsp10 protein as a potential target to develop better diagnostic and research reagents. PRRSV Nsp10 is one of the most conserved proteins encoded by ORF1b, a relatively conserved region among all the ORFs of PRRSV genome [5], and shares about 88% and 94% amino acid identity within genotype 1 and genotype 2 PRRSV respectively. Further, the presence of Nsp10 is a definite indication of virus infection, since PRRSV Nsp10 is exclusively synthesized during PRRSV replication cycles and do not incorporate into viral particles. In addition, Nsp10 is a viral helicase required for virus replication and genomic transcription [10]. Thus, Nsp10 protein may represent a novel target for PRRSV antivirals.

Monoclonal antibodies (mAb) are useful and effective tools in diagnostics and fundamental research on several aspects, especially for mapping antigenic epitopes of viral proteins. Therefore, we generated a PRRSV Nsp10-specific mAb 4D9 and mapped the epitope of mAb 4D9 in this study.

For PRRSV, B-cell epitopes mapping were mainly focused on the structural proteins, including GP3, GP5, M and N protein [16–19]. However, B-cell epitopes mapping in non-structural proteins (Nsps) were conducted only in Nsp1 and Nsp2 [20, 21]. Although there have been reports on the preparation PRRSV genotype-specific mAbs [22], this is the first Nsp10 mAb produced successfully. By using western blotting and IFA, we proved that the motif ^286^AIQPDYRDKL^295^ is the shortest epitope of mAb 4D9, and is a linear B-cell epitope. PRRSV-specific mAb that recognizes different antigenic isolates is particularly important for reliable PRRSV diagnostics. The mAb 4D9 generated in this study could recognize genotype 2 PRRSV isolate but not genotype 1 PRRSV isolate, suggesting that the mAb 4D9 may serve to differentiate between genotype 2 and genotype 1 PRRSV isolates. The mAb 4D9 coupled with Nsp10 protein can be used to develop a blocking ELISA to differentiate antibodies produced by pigs immunized with modified-live vaccines or killed vaccines. Currently, serological testing still remains a reliable tool for the diagnosis of PRRSV infection [23]. Several commercial ELISA Kits for the detection of antibodies against PRRSV in serum are available in the market [24, 25]. Each method has its advantages and drawbacks. Here, we showed the possibility of using the mAb 4D9 to develop an alternative method. Moreover, we have proved that the mAb 4D9 could specifically recognize both eukaryotic expressed Nsp10 protein and the viral Nsp10 protein from PRRSV-infected cells by confocal microscopy and Western blotting analysis, indicating that the mAb 4D9 is helpful for basic research focusing on the biological functions of Nsp10.

## Conclusions

In summary, we established a hybridoma that secrets genotype 2 PRRSV Nsp10-specific mAb 4D9, and defined a novel linear B-cell epitope on Nsp10 of PRRSV. Potentially, mAb 4D9 may be utilized for the PRRSV genotype-specific diagnosis and the study of PRRSV Nsp10 function.

## Methods

### Cells and viruses

The Marc-145 cell line (an African green monkey kidney epithelial cell line), human embryonic kidney HEK 293FT cell line, myeloma cell line SP2/0 and Vero cell line were maintained in Dulbecco’s modified Eagle’s medium (DMEM) supplemented with 10% fetal bovine serum at 37 °C with 5% CO_2_. The genotype 2 PRRSV strain CHsx1401 [26], and the genotype 1 PRRSV strain GZ11-G1 [27], which were described previously, were used in this study.

### Plasmid construction

Part of Nsp10 gene (from 433 to 1131 nucleotide in Nsp10) of PRRSV BJ-4 isolate (No. AF331831) was cloned into prokaryotic expression vector pET-28a via *BamH I/Hind III* sites to generate a recombinant plasmid pET-28a-Nsp10, which was constructed previously in our laboratory. The eukaryotic plasmids expressing HA-tagged or GFP-tagged Nsp10 and its truncation derivatives were made by cloning the corresponding gene fragments into the *EcoR*I and *Kpn*I clone site of eukaryotic vectors pCMV-HA and pEGFP-C1 respectively. The primers used in this study are listed in Table 1. The pEGFP-Nsp10 (D290C/D293E) plasmid that expressing point mutation mutant of the identified epitope was generated by inserting the synthesized gene into a pEGFP-C1 vector plasmid (Clontech). All recombinant plasmids used in this study were verified by DNA sequencing.

**Table 1:** Primers used for amplifying Nsp10 gene fragments and the corresponding amino acid sequence and size of each fragment

Name		Primer sequence (5′-3′)	Amino acid (aa) sequence	Size (aa)
A-1	F	GAATTCTGGGAAGAAGTCCAGAATG	^1^GKKSR^……^QTRRG^100^	100
	R	GGTACCTCATCCGCGGCGAGTCTGG		
A-2	F	GAATTCTTTAGTCTCCGTTAGGCG	^101^LVSVR^……^TLQFP^200^	100
	R	GGTACCTCAAGGGAATTGCAGCGTTG		
A-3	F	GAATTCTGCCCCCTCCCGTACCGG	^201^APSRT^……^MSMVN^300^	100
	R	GGTACCTCAGTTGACCATGGACATAAG		
A-4	F	GAATTCTACGACCCGTGTGACCTAC	^301^TTRVT^……^CADLE^441^	141
	R	GGTACCTCATTCCAGGTCTGCGCAAATAG		
B-1	F	GAATTCTGCCCCCTCCCGTACCGG	^201^APSRT^……^LRLLS^240^	40
	R	GGTACCTCAACTGAGAAGCCTCAAG		
B-2	F	GAATTCTAATCACCTTGATGTCTTG	^231^NHLDV^……^DIMPQ^270^	40
	R	GGTACCTCACTGAGGCATGATGTCA		
B-3	F	GAATTCTCATTGCTATGTATTTGAC	^261^HCYVF^……^MSMVN^300^	40
	R	GGTACCTCAGTTGACCATGGACATAAG		
C-1	F	GAATTCTCATTGCTATGTATTTGAC	^261^HCYVF^……^IWRFG^280^	20
	R	GGTACCTCACCCGAACCTCCAGATGG		
C-2	F	GAATTCTACCCAATTAAAGACCATC	^271^TQLKT^……^AIQPD^290^	20
	R	GGTACCTCAATCTGGTTGAATGGCATC		
C-3	F	GAATTCTCAGAATATCTGTGATGC	^281^QNICD^……^MSMVN^300^	20
	R	GGTACCTCAGTTGACCATGGACATAAG		
D-1	F	GAATTCTCAGAATA…CAAACTTTGAGGTACC	^281^QNICDAIQPDYRDKL^295^	15
	R	GGTACCTCAAAGTTTG…TATTCTGAGAATTC		
D-2	F	GAATTCTGCCATTC…GGTCAACTGAGGTACC	^286^AIQPDYRDKLMSMVN^300^	15
	R	GGTACCTCAGTTGACC…GAATGGCAGAATTC		
E-1	F	GAATTCTGCC…GAT…GAC…CTTTGAGGTACC	^286^AIQPDYRDKL^295^	10
	R	GGTACCTCAAAG…GTC…ATC…GGCAGAATTC		
E-1- mutant	F	GAATTCTGCC…TGT…GAG…CTTTGAGGTACC	^286^AIQPCYREKL^295^	10
	R	GGTACCTCAAAG…CTC…ACA…GGCAGAATTC		
E-2	F	GAATTCTGCCATTCAA…GACAAATGAGGTACC	^286^AIQPDYRDK^294^	9
	R	GGTACCTCATTTGTC...TTGAATGGCAGAATTC		
E-3	F	GAATTCTATTCAA…GACAAACTTTGAGGTACC	^287^IQPDYRDKL^295^	9
	R	GGTACCTCAAAGTTTGTC...TTGAATAGAATTC		

The forward (F) and reverse (R) primers contain *EcoR*I and *kpn*I recognition sites, respectively (underlined). The superscript numbers indicate the relative location of the amino acids in Nsp10 protein

### Prokaryotic expression and purification of recombinant PRRSV Nsp10 protein

The prokaryotic expression plasmid pET-28a-Nsp10 was transformed into *E. coli* BL21 (DE3) competent cells. The Nsp10 expression was induced by adding isopropyl β-D-1-thiogalactopyranoside (IPTG) to the medium containing the transformed bacteria at a final concentration of 1 mM. The expressed fusion protein was analyzed by SDS-polyacrylamide gel electrophoresis (SDS-PAGE) and visualized using Coomassie brilliant blue staining. Purification of recombinant Nsp10 was carried out by excising the gel piece containing Nsp10 after SDS-PAGE and further dialyzing Nsp10 out of the gel piece. Western blotting was employed to analyze the antigenicity and purity of the purified protein.

### Generation of Nsp10-specific mAb

To prepare mAb against PRRSV Nsp10, 6- to 8-week-old BALB/c mice were subcutaneously immunized with 100 μg purified recombinant Nsp10 protein emulsified with an equal volume of Freund’s complete adjuvant (Sigma). Two booster immunizations of incomplete Freund’s adjuvant (Sigma) emulsified antigen were given at two-week intervals with the same dose. Two weeks after the third immunization, the mice were intraperitoneally boosted with purified Nsp10 alone. Three days later, the spleen cells from the immunized mice were fused with myeloma cells SP2/0, and hybridoma supernatants were screened by indirect enzyme-linked immunosorbent assay (ELISA) as described below. The hybridoma cells tested positive in the ELISA were subcloned three times by limiting dilution, and the supernatants from those cells were further characterized by Western blotting and confocal microscopy. Ascitic fluid was prepared by intraperitoneal injection of BALB/c mice with the produced hybridoma cells. A commercial IsoQuick™ Strips and Kits for Mouse Monoclonal Isotyping (ISOQ5-1KT, EnviroLogix Inc.) was used to determine the mAb subtypes. Antibodies isotyping was performed according to manufacturer’s instructions.

### Indirect ELISA

Ninety-six-well microtiter plates were coated with the purified Nsp10 protein in 0.1 M carbonate- bicarbonate buffer (pH 9.6) at 4 °C overnight and then blocked with 5% skim milk for 1 h at 37 °C. After washing three times with 0.1% Tween-20 in phosphate buffered saline (PBS), the plates were incubated with 100 μl of hybridoma supernatants at 37 °C for 1 h. Subsequently, the plates were washed thrice and incubated with horseradish peroxidase (HRP)-conjugated goat anti-mouse IgG (ZSGB-BIO, China) at a dilution of 1:10,000 in PBS at 37 °C for 1 h. A substrate solution containing o-phenylenediamine (OPD) was added for color development and the enzymatic reaction was stopped with 2 M H_2_SO_4_. The absorbance was measured at 490 nm using a Microplate Reader.

### Western blotting

After being separated by SDS-PAGE, protein samples were electrically transferred onto a polyvinylidene fluoride (PVDF) membrane. Then, the membrane was blocked with 5% skim milk in PBS for 2 h at room temperature (RT), followed by incubation with appropriate primary antibodies. After three washes with PBST, the membrane was incubated with proper horseradish peroxidase (HRP)-conjugated secondary antibodies at 37 °C for 1 h. The membrane was washed again and developed with the ECL Western blotting system (Pierce).

### Confocal microscopy

The procedure for confocal microscopy has been described previously [28]. Briefly, Vero cells grown on coverslips in six-well plates at about 50% confluence were transfected with plasmids expressing Nsp10. At 24 h post-transfection, the cells were fixed with 3.7% paraformaldehyde for 10 min at RT, and permeabilized with PBS containing 0.1% Triton X-100 and 2% BSA for 10 min, then blocked with 2% BSA/PBS for 30 min. Nsp10 was stained with Nsp10-specific mAb 4D9 in a humid chamber for 1 h at RT. After three times of 5 min washing with PBS, the cells were then incubated with Alexa Fluor 488-conjugated goat anti-mouse IgG F(ab’)_2_ fragment (Molecular Probes) secondary antibody for 1 h at RT. Nuclear DNA was stained with 4′,6-diamidino-2-phenylindole (DAPI) (Molecular Probes) for 5 min at RT. The images were collected with an Olympus confocal microscope. Alternatively, MARC-145 cells were infected with PRRSV strains CHsx1401or GZ11-G1 and the cells were fixed with 3.7% paraformaldehyde at 12 h post-infection (hpi) and were stained as described above.

### Mapping of the mAb 4D9 epitope in Nsp10

To primarily locate the mAb epitope containing region, full-length Nsp10 was divided into four fragments of different lengths. Schematic representation of Nsp10 fragments used for epitope mapping are shown in Fig. 3. The different fragments of Nsp10 were analyzed with Western blotting using anti-GFP polyclonal antibodies and Nsp10-specific mAb. The fragment that reacted with both Nsp10-specfic mAb and anti-GFP antibodies was identified as the epitope-containing region. The identified fragment was finely mapped by testing the reaction of Nsp10-specific mAb 4D9 with a serial peptides truncated one by one from the N- and C-termini of the identified fragment respectively (Fig. 3). The smallest peptide recognized by Nsp10-specific mAb 4D9 was defined as the mAb’s epitope.

### Homology analysis

To investigate the homology of the mAb 4D9 epitope among PRRSV viruses, we performed a sequence alignment with Nsp10 gene of both genotype 1 and genotype 2 PRRSV deposited in GenBank using the MegAlign software. Based on the sequence alignment, a representative mutant motif, AIQPCYREKL, was designed and expressed as a GFP-fusion protein to test whether two amino acid substitutions in the epitope affect the reactivity between Nsp10-specific mAb and the epitope.

### Reactivity of Nsp10-specific mAb with different genotypes of PRRSV isolates

Two representative strains of genotype 1 and genotype 2 PRRSV, GZ11-G1 and CHsx1401, were used to determine their reactivity with Nsp10-specific mAb 4D9 by Western blotting and confocal microscopy. In brief, MARC-145 cells were infected with GZ11-G1 or CHsx1401, respectively. The virus infected cells were harvested for Western blotting analysis at 36 hpi or confocal microscopy at 12 hpi respectively.

## Abbreviations

ELISA: indirect enzyme-linked immunosorbent assay
Hpi: h post-infection
IPTG: isopropyl β-D-1-thiogalactopyranoside
mAb: monoclonal antibody
Nsp: non-structural protein
ORF: open reading frame
PBS: phosphate buffered saline
PRRSV: porcine reproductive and respiratory syndrome virus
RT: room temperature
